# Effects of lifestyle modification in polycystic ovary syndrome compared to metformin only or metformin addition: A systematic review and meta-analysis

**DOI:** 10.1038/s41598-020-64776-w

**Published:** 2020-05-08

**Authors:** Chan Hee Kim, Seung Joo Chon, Seon Heui Lee

**Affiliations:** 1 Graduate student for Master of Nursing, Gachon University, 191, Hambangmoe-ro, Yeonsu-gu, Incheon, 21936 Incheon, Republic of Korea; 2 Department of Obstetrics and Gynecology, Gil Hospital, Gachon University College of Medicine, Namdongdae-ro 774, Namdong-gu, Incheon, 21565 Republic of Korea; 3 Department of Nursing Science, College of Nursing, Gachon University, 191, Hambangmoe-ro, Yeonsu-gu, Incheon, 21936 Republic of Korea

**Keywords:** Infertility, Weight management

## Abstract

Polycystic ovary syndrome (PCOS) is a common disease that has an effect on approximately 10% of women of childbearing age. Although there is evidence regarding the role of lifestyle factors in the development of PCOS, the exact etiology remains unclear. Additionally, metformin is used in the treatment of PCOS but its role remains unclear. We compared the effects of lifestyle modification (LSM) + metformin and metformin alone on PCOS. We performed a systematic review by searching electronic databases for publications until December 2019. The primary endpoints were clinical outcomes, such as menstrual cycles and pregnancy rates, and the secondary endpoints were anthropometric, metabolic, and androgenic parameters. The meta-analysis revealed that there was no significant difference in the improvements in the menstrual cycles between LSM and metformin alone (weighted mean difference [MD] = 1.62) and between LSM + metformin and LSM (MD = 1.20). The pregnancy rates and body mass indices were not significantly different between LSM and metformin alone (MD = 1.44 and −0.11, respectively). LSM reduced insulin resistance (MD = −0.52) and increased serum levels of sex hormone-binding globulins (MD = 8.27) compared with metformin. Therefore, we suggest recommending lifestyle modifications actively to women with PCOS if they do not have indications for metformin.

## Introduction

Polycystic ovary syndrome (PCOS) is a common disease that has an effect on approximately 10% of women of child bearing age^1^. The Rotterdam criteria (ratified by the Australian PCOS Alliance and the US National Institutes of Health [NIH]) are internationally recognized and used in diagnosing PCOS, which requires two of the following three features: excess androgens, ovulatory dysfunction, and polycystic ovarian morphology^2^. Other significant manifestations include metabolic abnormalities, such as insulin resistance, dyslipidemia, and type II diabetes.

Although the etiology of PCOS is still unclear, it certainly is a multifactorial disorder, and it appears to be associated with biochemical abnormalities and pro-inflammatory metabolic imbalance^3–7^. Recent studies have demonstrated that obesity and PCOS are interrelated; obesity increases the prevalence of PCOS and PCOS results in weight gain and obesity^8^. Insulin resistance, hyperandrogenism, and the severity of PCOS can be improved through lifestyle modification (LSM), such as dietary modifications, physical exercises, or behavioral changes, medications, such as metformin, or bariatric surgery^9,10^. Weight loss has positive effects on the clinical improvement in menstrual function, fertility^11^, pregnancy outcomes, and endocrine parameters^12^. However, the efficacy of LSM for PCOS varies based on the type of lifestyle management and characteristics of PCOS^13^. Women with PCOS who are overweight or obese are expected to benefit from LSM that result in adiposity reduction^14^ and ovulation^15^; however, it remains unclear if LSM is also efficacious in women with PCOS having normal weight^16^.

A systematic review is needed to evaluate the effects of therapies in the management of PCOS. Several systematic reviews have confirmed the efficacy of LSM and metformin for PCOS, which result in improvements in body weight, insulin resistance, hyperandrogenism, and ovulation^17,18^. Although a previous systematic review has compared the effects of LSM + metformin with those of LSM + placebo on PCOS, there were some errors; the baseline data were used instead of the final result data^18^, which resulted in a significant effect of metformin. The purpose of this review was to assess the effects of LSM on PCOS, compare them with those of metformin on PCOS, and investigate if the combination of LSM + metformin is more effective than either of them alone against PCOS.

## Results

### Study selection

**Figure 1:**
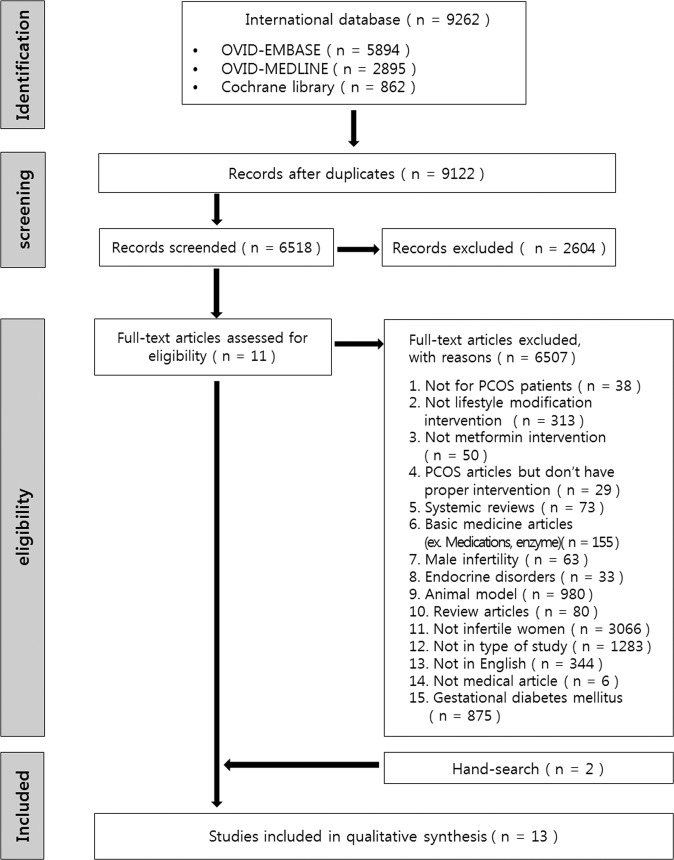
Study flow chart.

After a full-text review, 11 articles were identified as relevant for this study. Two additional articles were identified by manually searching the relevant bibliographies, and 13 publications were finally included in the meta-analysis (Fig. 1).

### Characteristics of included studies

Table 1 summarizes the characteristics of the 13 studies, which included 11 randomized controlled trials (RCTs), one clinical trial, and one prospective study. These studies were published between 2000 and 2018 and included four studies from Europe, four studies from North America, two studies from South America, two studies from the Middle East, and one study from Africa. These studies were divided based on the comparisons made in them; six studies compared LSM with metformin alone, eight studies compared LSM + metformin with LSM, and one study was included in both.

**Table 1:** Characteristics of included studies.

Study and year	Country	Study design	Definition of PCOS	Group	Intervention	No.	Age, y	F-G score	Reproductive outcome index
**LSM vs. LSM + MET**									
Ladson(1) (2011)	USA	RCT	1990 NIH/NICHD PCOS diagnostic criteria^36^	LSM	Combined: hypocaloric diet (500 kcal of deficit) and aerobic exercise (150 m/week) for 6 months Placebo: one capsule/d	10	15.4 ±1.2		- Ovarian volume - Maximum follicle size - No. of menstrual bleeding
				LSM + MET	Combined: same as LSM Metformin: 500 mg/d gradually increased to 2 g/day, every 5 days)	10	16.1 ±1.5		
Ladson(2) (2011)	USA	RCT	1990 NIH/NICHD PCOS diagnostic criteria	LSM	Combined: hypocaloric diet (500 kcal of deficit) and aerobic exercise (150 m/week) for 6 months Placebo: one capsule/d	59	28.8 ±4.6	19.1 ±8.9	- Ovulation rate
				LSM + MET	Combined: same as LSM Metformin: 500 mg/d gradually increased to 2 g/day, every 5 days)	55	29.0 ±4.5		
Otta (2010)	Argentina	RCT	Hyperandrogenism and oligomenorrhea or amenorrhea	LSM	Combined: diet (1500 kcal/d) and exercise (walking 40 m/d, 4 d/week) for 4 months Placebo	15	24.7 ±3.46	13.5 ±5.97	- Menstrual cycle regulation - Ovulation rate
				LSM + MET	Combined: same as LSM Metformin: 500 mg/d on 1^st^ week, 500 mg*2/d on 2^nd^ week, 750 mg*2/d on 3^rd^ week	15	25.47 ±4.82	11.73 ±5.31	
Tang (2006)	UK	RCT	Presence of polycystic ovaries on transvaginal scan and oligomenorrhea or amenorrhea	LSM	Combined: hypocaloric diet (500 kcal of deficit) and daily exercise (walking 15 min/d) Placebo: one tablet*2/d for 6 months	74	29.8 ±3.8		- Pregnancy rate - Menstrual cycle regulation
				LSM + MET	Combined: same as LSM Metformin: 850 mg*2/d for 6 months	69	29.7 ±3.7		
Hoeger (2004)	USA	RCT	Hyperandrogenism and oligomenorrhea or amenorrhea	LSM	Combined: diet (500–1000 kcal/d) and exercise (150 min/week) for 12 months Placebo: one tablet*2/d	11	27.1 ±4.3		- No. of documented ovulation - No. of reported menstrual events
				LSM + MET	Combined: same as LSM Metformin: 850 mg*2/d	9	30.4 ±5.4		
Salama (2018)	Egypt	Clinical trial	Rotterdam 2003 criteria	LSM	Combined: hypocaloric diet and physical activity for 12 weeks	75	20–40		- No. of patients with improvement in menses - Pregnancy rate
				LSM + MET	Combined: same as LSM Metformin: 850 mg*2/d	75	20–40		
**Diet vs. Diet +MET**									
Gambineri (2006)	Italy	RCT	Rotterdam 2003 criteria	Diet	Diet: diet (1200 and 1400 kcal/d) Placebo: 1 tablet*2/d	20	26 ±5	9.3 ±4.8	- Frequency of menstruation
				Diet +MET	Diet: same as Diet Metformin: 850 mg*2/d and Diet	20	28 ±8	13 ±8.9	
Pasquali (2000)	Italy	RCT	Hyperandrogenism and Oligomenorrhea or Amenorrhea	Diet	Diet: hypocaloric diet (1200–1400 kcal/d) Placebo: 1 tablet*2/d for 6 months	8	32.3 ±5.0		- Frequency of menstruation
				Diet +MET	Diet: same as Diet Metformin: 850 mg*2/d for 6 months	12	30.8 ±7.4		
**LSM vs. MET**									
Curi (2012)	Brazil	RCT	Rotterdam 2003 criteria	LSM	Combined: diet (500 kcal of deficit) and exercise (walking 40 m/d, 4 d/week) for 6 months	15	24.6 ±1.3	15.7 ±1.56	- Menstrual cycle index (frequency of menstruation)
				MET	Metformin: 850 mg*2/d for 6 months	12	26.3 ±1.4	13.17 ±1.74	
Karimzadeh (2010)	Iran	RCT	Rotterdam 2003 criteria	LSM	Combined: hypocaloric diet (500 cal of deficit) and exercise (120 min/d, 3–5 d/week) for 6 months	75	27.34 ±2.27		- Clinical pregnancy rate - Multiple pregnancy rate - Menstrual cycle regulation
				MET	Metformin: 1,50 0 mg/d for 3–6 months	90	27.33 ±2.34		
Hoeger (2008)	USA	RCT	Hyperandrogenism and menstrual irregularity	LSM	Combined: diet (500–1000 kcal/d) and exercise (150 min/week) for 12 months	11	15.4 ±1.2	9.1 ±1.5	- Average of Menstrual cycles per 24 wk
				MET	Metformin: 1700 mg/d for 12 months	10	16 ±1.7	7.8 ±3.1	
Hoeger (2004)	USA	RCT	Hyperandrogenism and oligomenorrhea or amenorrhea	LSM	Combined: diet (500–1000 kcal/d) and exercise (150 min/week) for 12 months Placebo: one tablet*2/d	11	27.1 ±4.3		- No. of documented ovulation- No. of reported menstrual events
				MET	Metformin: 850 mg*2/d	9	29.5 ±6.4		
**Diet vs. MET**									
Esfahanian (2013)	Iran	RCT	Rotterdam 2003 criteria	Diet	Diet: hypocaloric diet for 5%–10% weight loss for 12 weeks	17	21.9 ±9.3		- No. of patients with improvement in menses - Menstrual quantity
				MET	Metformin: 500 mg*2/d for 12 weeks	13	20 ±4.6		
Qublan (2007)	Greece	Prospective	Rotterdam 2003 criteria	Diet	Diet: 1200–1400 kcal/d for 6 months	24	31.5	15	- Pregnancy rate - Menstrual cycle regulation - Ovulation rate
				MET	Metformin: 850 mg*2/d for 6 months	22	30.8	16	

BMI: body mass index, CO: control, Ex: exercise, F-G score: Ferriman-Gallwey score, LSM: lifestyle modification (diet+exercise), MET: metformin, No.: number of patients, OC: oral contraceptive pills, PCOS: polycystic ovary syndrome, RCT: randomized controlled trial, WHR: waist-hip ratio.

### Risk of bias

**Figure 2:**
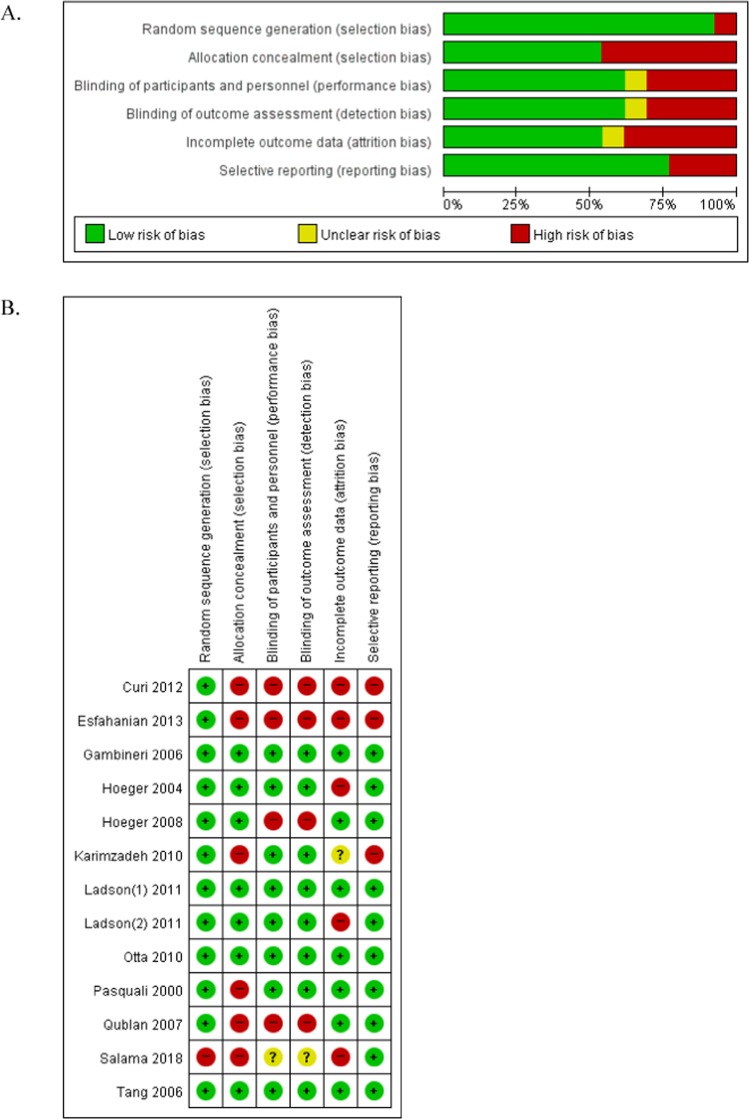
Assessment of the risk of bias. (**A**) Risk of bias graph; (**B**) Risk of bias summary.

The assessment of the risk of bias within these studies revealed the following results. Six studies had a high risk of selection bias, and four studies reported a high risk in the blinding of participants, personnel, and outcome assessments. Additionally, five studies had a high risk of attrition bias associated with high drop rates (Fig. 2).

### Clinical outcomes

#### Menstrual cycle

**Figure 3:**
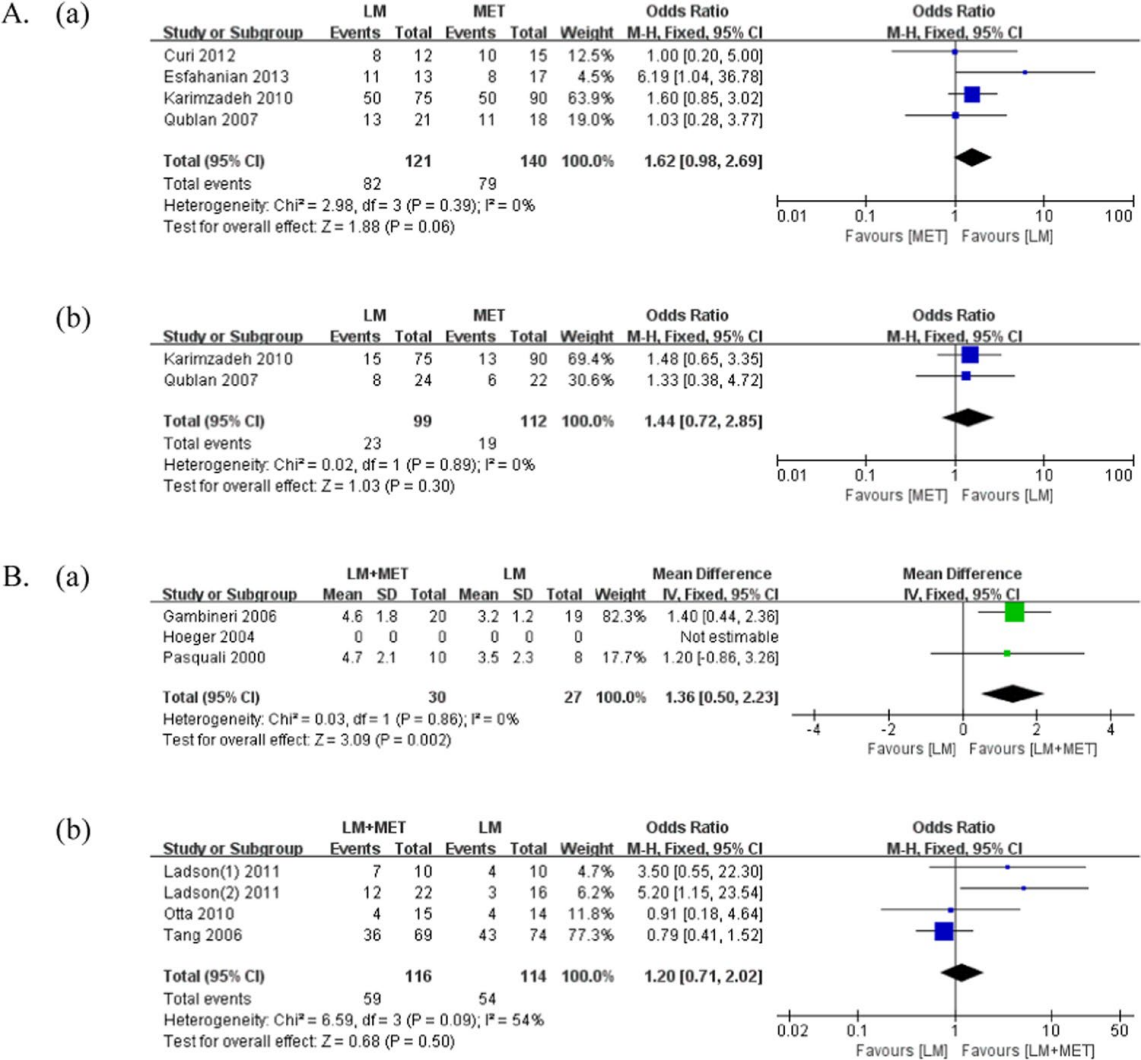
Meta-analysis of the clinical outcomes. (**A**) Lifestyle modifications versus metformin alone (**a**) Number of patients with improvement in menstrual frequency **(b**) Pregnancy rate; (**B**) Lifestyle modifications + metformin versus lifestyle modifications (**a**) Number of menstrual cycles over 6 months (**b**) Number of patients with improvement in menstrual frequency.

A comparison of the clinical outcomes is presented in Table 2 and Fig. 3. Menstrual cycles were evaluated in all studies that compared LSM with metformin alone; four studies were included for meta-analysis. There was no significant difference in the improvement in the **menstrual** frequency between the groups (p = 0.06; weighted mean difference [MD] = 1.62). However, two studies reported trends of improvement in the menstrual frequency with LSM compared with metformin alone (Karimzadeh *et al*.^19^: 66.6% vs. 55.5%; Esfahanian *et al*.^20^: 84.6% vs. 47.1%).

**Table 2:** Differences in clinical outcomes before, after, and during the interventions.

Author Year	Group	Weight				BMI				WHR			Menstrual cycle regularity			Pregnancy rate	
		Before	After		p	Before	After		p	Before	After	p	Index	Events/Total (%)	p	Events/Total (%)	p
**LSM vs. LSM + MET**																	
Ladson(1) 2011	LSM					38.3 ±8.0							No. of menstrual bleeding	LSM + MET vs. LSM	NS		
	LSM + MET					38.0 ±7.8								RR = 1.7 95% CI = 0.7–3.9			
Ladson(2) 2011	LSM	Mean change	−1.6	⇓	0.001								Ovulation rate (urinary pregnanediol)	LSM + MET vs. LSM	NS		
	LSM + MET	Mean change	−2.1	⇓	<0.001									RR = 2.5 95% CI 0.9–6.6			
Otta 2010	LSM					35.6 ±4.98	34.16 ±4.95		NS	0.91	0.92	NS	No. of patients in regular menstrual cycling	4/14 (28.5%)	NR		
	LSM + MET					32.4 ±6.7	31.53 ±4.98		NS	0.88	0.85	NS		4/15 (26.6%)			
Tang 2006	LSM	100.7 ±17.9	99.2 ±17.3	⇓	<0.1	37.9 ±6.5	37.4 ±6.3	⇓	<0.05	0.894 ±0.15	0.899 ±0.097	NS	No. of patients in regular menstrual cycling	43/74 (58.1%)	NS	2/74 (2.7%)	NS
	LSM + MET	102.7 ±15.0	99.9 ±15.0	⇓	<0.001	38.1 ±5.08	37.1 ±5.04	⇓	<0.01	0.906 ±0.094	0.911 ±0.098	NS		36/69 (52.2%)		6/69 (8.6%)	
Hoeger 2004	LSM	Percent change	−6.8 ±3.8	⇓	<0.05								Mean no. of documented ovulation (urinary pregnanediol)	3.5	NR		
	LSM + MET	Percent change	−8.9 ±2.9	⇓	<0.05									3.2			
Salama 2018	LSM	Percent change	−7.15 ±3.30	⇓	<0.05	Percent change	−7.13 ±3.33	⇓	<0.05	Percent change	−2.15 ±2.78	<0.05	No. of patients with improvement in menses (12 weeks)	27/43 (62.8%)	NS	7/58 (12.0%)	NS
	LSM + MET	Percent change	−6.37 ±2.85	⇓	<0.05	Percent change	−6.37 ±2.87	⇓	<0.05	Percent change	−3.11 ±3.41	<0.05		25/40 (62.5%)		6/51 (11.8%)	
**Diet vs. Diet + MET**																	
Gambineri 2006	Diet	97 ±16	92 ±16	⇓	<0.001	37 ±5	35 ±5	⇓	<0.01				No. of menses in 6 months	2.7 ±1.2	3.2 ±1.2	<0.05	NR
	Diet +MET	92 ±13	88 ±13	⇓	<0.01	35 ±4	33 ±5	⇓	<0.01					2.6 ±1.6	4.6 ±1.8	<0.001	
Pasquali 2000	Diet	102 ±19	97 ±18	⇓	<0.01	39.6 ±6.9	38.0 ±6.2	⇓	<0.05	0.91 ±0.11	0.88 ±0.05	NS	No. of menses in 6 months	1.3 ±1.5	3.5 ±2.3	<0.05	<0.05
	Diet +MET	103 ±18	94 ±17	⇓	<0.001	39.8 ±7.9	36.4 ±7.4	⇓	<0.001	0.87 ±0.07	0.86 ±0.07	NS		1.2 ±1.6	4.7 ±2.1	<0.01	
**LSM vs. MET**																	
Curi 2012	LSM					31.8 ±1.6	30.1 ±1.5	⇓	<0.01				No. of patients with improvement in menses	8/12 (66.6%)	NS		
	MET					31.4 ±1.4	30.2 ±0.8	⇓	<0.05					10/15 (66.6%)			
Karimzadeh 2010	LSM					27.92 ±1.05							No. of patients with improvement in menses	50/75 (66.6%)	NS	15/75 (20.0%)	NS
	MET					27.17 ±1.73								50/90 (55.5%)		13/90 (14.4%)	
Hoeger 2008	LSM					36 ±6.2	34.9 ±7		NS				Average of menstrual cycle per 24 weeks	2.3	NS		
	MET					35 ±8.2	35.7 ±8.6		NS					3.2			
Hoeger 2004	LSM	Percent change	−6.8 ±3.8	⇓	<0.05								Mean no. of documented ovulation (urinary pregnanediol)	3.5	NR		
	MET	Percent change	−6.5 ±3.7	⇓	<0.05									4.3			
**Diet vs. MET**																	
Esfahanian 2013	Diet					34.1 ±5.4	30.1 ±5.5	⇓	<0.001	0.8 ±0.04	0.7 ±0.05	0.01	No. of patients with improvement in menses	11/13 (84.6%)	NR		
	MET					31.1 ±3.3	30.3 ±3.5	⇓	<0.001	0.78 ±0.05	0.77 ±0.05	NS		8/17 (47.1%)			
Qublan 2007	Diet					32.2	27.4	⇓	<0.01				No. of patients in regular menstrual cycling	13/21 (61.9%)	NS	8/24 (33.3%)	NS
	MET					31.9	27.8	⇓	<0.01					11/18 (61.1%)		6/22 (27.3%)	

BMI: body mass index, LSM: lifestyle modification, MET: metformin, NR: not response, NS: no significance, WHR: waist-hip ratio.

Menstrual cycles were evaluated in all studies that compared LSM + metformin with LSM. We conducted two types of meta-analysis regarding menstrual cycles. First, we compared the number of menstrual cycles over 6 months between the groups; for this purpose, two studies were included in the meta-analysis. LSM + metformin resulted in a significantly greater number of menstrual cycles over 6 months than LSM alone (p < 0.01, MD = 1.36). In all included studies, both groups demonstrated a significant increase in the number of menstrual cycles over 6 months. Second, we compared the number of patients with improvement in the menstrual frequency between the groups; for this purpose, four studies were included in the meta-analysis. There was no significant difference in the improvement in menstrual frequency between the groups (p = 0.50, MD = 1.20).

### Pregnancy rate

The pregnancy rate was evaluated in two studies that compared LSM with metformin alone. There was no significant difference in the pregnancy rate between the groups (p = 0.30, MD = 1.44).

The pregnancy rate was evaluated in two studies that compared LSM + metformin with LSM. In the study by Tang *et al*.^21^, the total pregnancy rates in the LSM (2.7%) group and LSM + metformin (8.7%) group were not significantly different (p = 0.233). Similarly, in the study by Salama *et al*.^22^, the total pregnancy rates in the LSM (12.0%) group and LSM + metformin (11.8%) group were not significantly different.

### Anthropometric parameters

#### Weight loss

**Figure 4:**
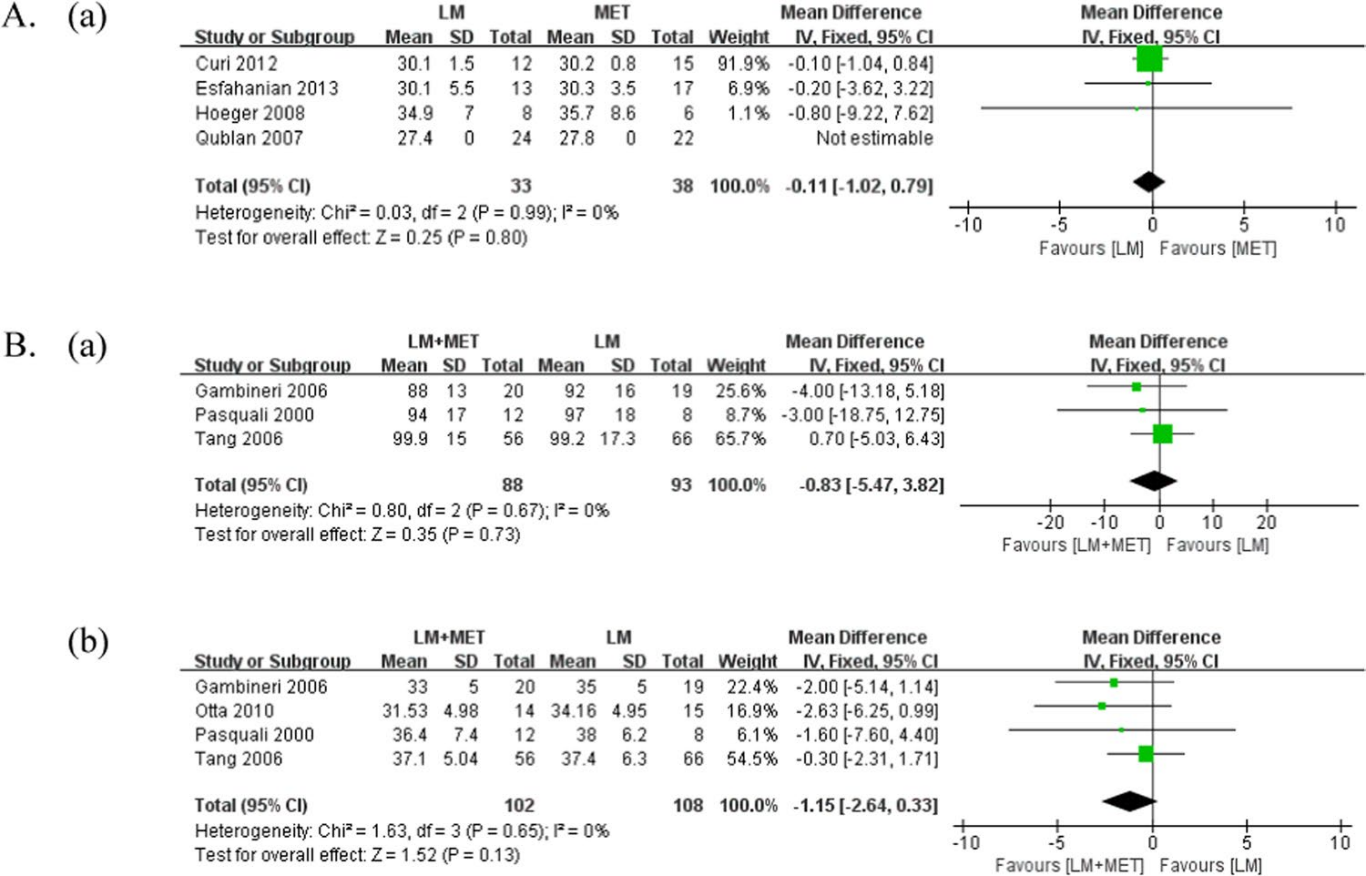
Meta-analysis of the anthropometric parameters. (**A**) Lifestyle modifications versus metformin alone (**a**) body mass index (BMI); (**B**) lifestyle modifications + metformin versus lifestyle modifications (**a**) Weight loss (**b**) BMI.

A comparison of the anthropometric parameters is presented in Table 2 and Fig. 4. Weight loss was measured in one study that compared LSM with metformin alone. Both LSM (percent change = −6.8 ± 3.8, p < 0.05) and metformin alone (percent change = −6.5 ± 3.7, p < 0.05) resulted in a significant amount of weight loss.

Weight loss was evaluated in six studies that compared LSM + metformin with LSM, and three studies were included in the meta-analysis. There was no significant difference in the weight loss between the groups (p = 0.73, MD = −0.83). All six included studies reported significant weight loss in both LSM + metformin and LSM groups.

#### Body mass index

Body mass index (BMI) was measured in five studies that compared LSM with metformin alone, and three studies were included for meta-analysis. There was no significant difference in BMI between the groups (p = 0.80, MD = −0.11). Three of five included studies reported a significant reduction in BMI in both LSM and metformin alone groups, while one study reported no significant difference in either group, and one study did not report the post-intervention data.

BMI was measured in six studies that compared LSM + metformin with LSM, and four studies were included for meta-analysis. LSM + metformin was associated with a lower BMI at study completion than LSM, whereas there was no significant difference between the groups in our study (p = 0.13, MD = −1.15). Four out of six studies reported a significant reduction in BMI in both groups, and one study did not find any significant reduction in either group. One study did not report the post-intervention data.

### Metabolic parameters

#### Fasting glucose

**Figure 5:**
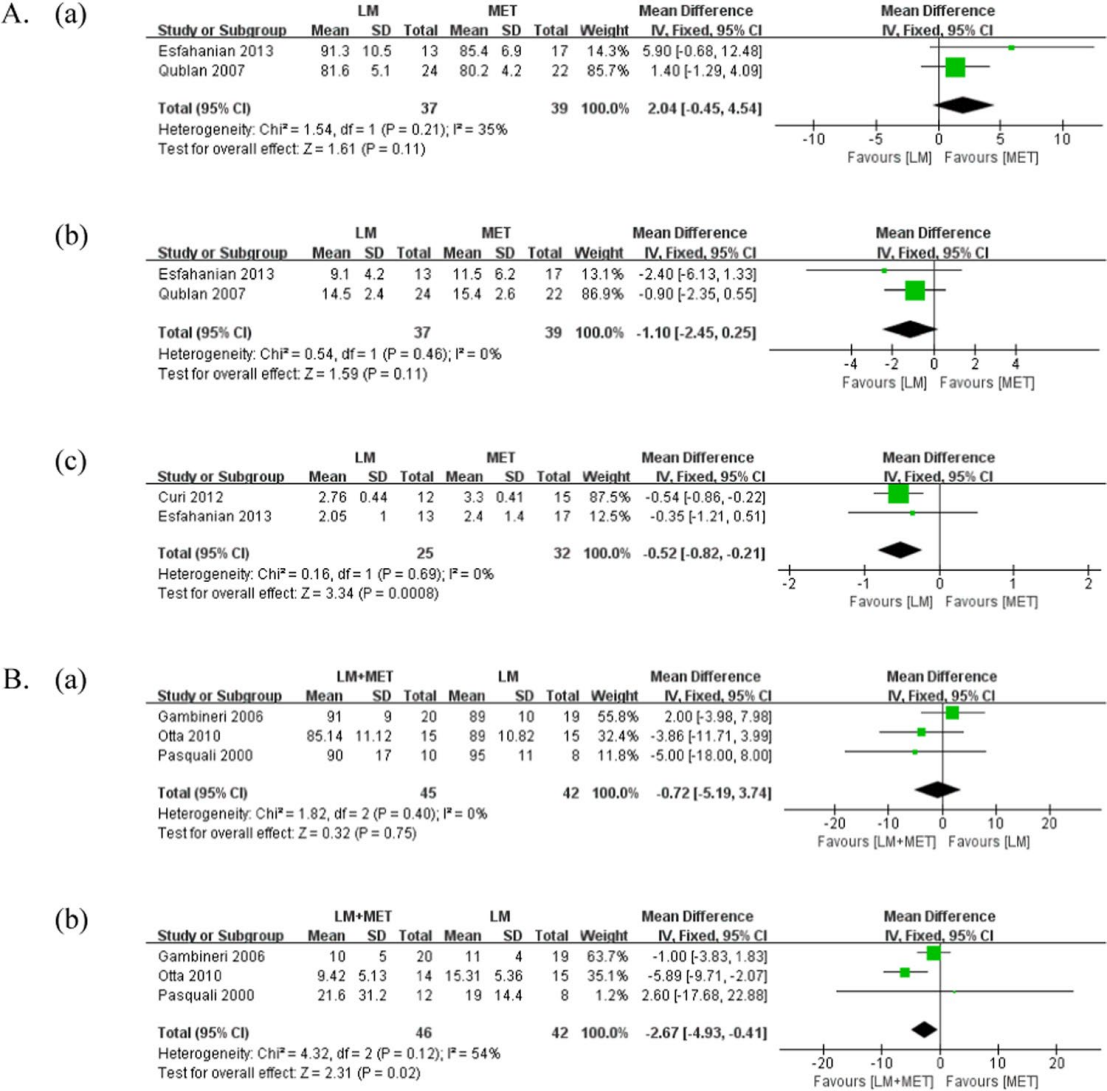
Meta-analysis of the metabolic parameters. (**A**) Lifestyle modifications versus metformin alone (**a**) Fasting serum glucose levels (**b)** Fasting serum insulin levels (**c**) Homeostatic model assessment for insulin resistance; (**B**) Lifestyle modifications + metformin versus lifestyle modifications (**a**) Fasting serum glucose levels (**b**) Fasting serum insulin levels.

A comparison of the metabolic parameters is presented in Fig. 5 and Supplementary 1. Fasting serum glucose level was measured in two studies that compared LSM with metformin alone. There was no significant difference in the serum fasting glucose level between the groups (p = 0.11, MD = 2.04). One study reported a significant reduction in fasting glucose with metformin alone, whereas the other study reported no significant difference in either group.

Fasting serum glucose level was measured in six studies that compared LSM + metformin with LSM, and three studies were included in the meta-analysis. There was no significant difference in the fasting serum glucose levels between the groups (p = 0.75, MD = −0.72). One out of five studies reported a significant reduction in fasting serum glucose levels with LSM + metformin; however, the majority of studies reported no significant differences in either group.

#### Fasting insulin

Fasting serum insulin level was measured in three studies that compared LSM with metformin alone, and two studies were included for meta-analysis. There was no significant difference in the fasting serum insulin levels between the groups (p = 0.11, MD = −1.10). One out of three studies reported a significant reduction in fasting serum insulin levels in both LSM and metformin groups, while two studies reported no significant difference in either group.

Fasting serum insulin level was measured in six studies that compared LSM + metformin with LSM, and three studies were included in the meta-analysis. LSM + metformin was reported to significantly reduce fasting serum insulin levels compared with LSM (p = 0.02, MD = −2.67). Three out of six studies reported a significant reduction in both groups, while one study reported a significant reduction with LSM + metformin alone, and two studies reported no significant differences in either group.

#### Homeostatic model assessment for insulin resistance

Homeostatic model assessment for insulin resistance (HOMA-IR) was measured in two studies that compared LSM with metformin alone. LSM was reported to significantly reduce HOMA-IR compared with metformin alone (p < 0.01; MD = −0.52). One out of two included studies reported a significant reduction in HOMA-IR in both LSM + metformin and metformin alone groups, while one study reported no significant difference in either group.

HOMA-IR was measured in one study that compared LSM + metformin with LSM, which reported that LSM + metformin significantly decreased HOMA-IR from 3.25 ± 1.11 to 2.06 ± 1.36 (p = 0.01), while there was no significant change in HOMA-IR with LSM.

### Androgenic parameters

#### Total testosterone

**Figure 6:**
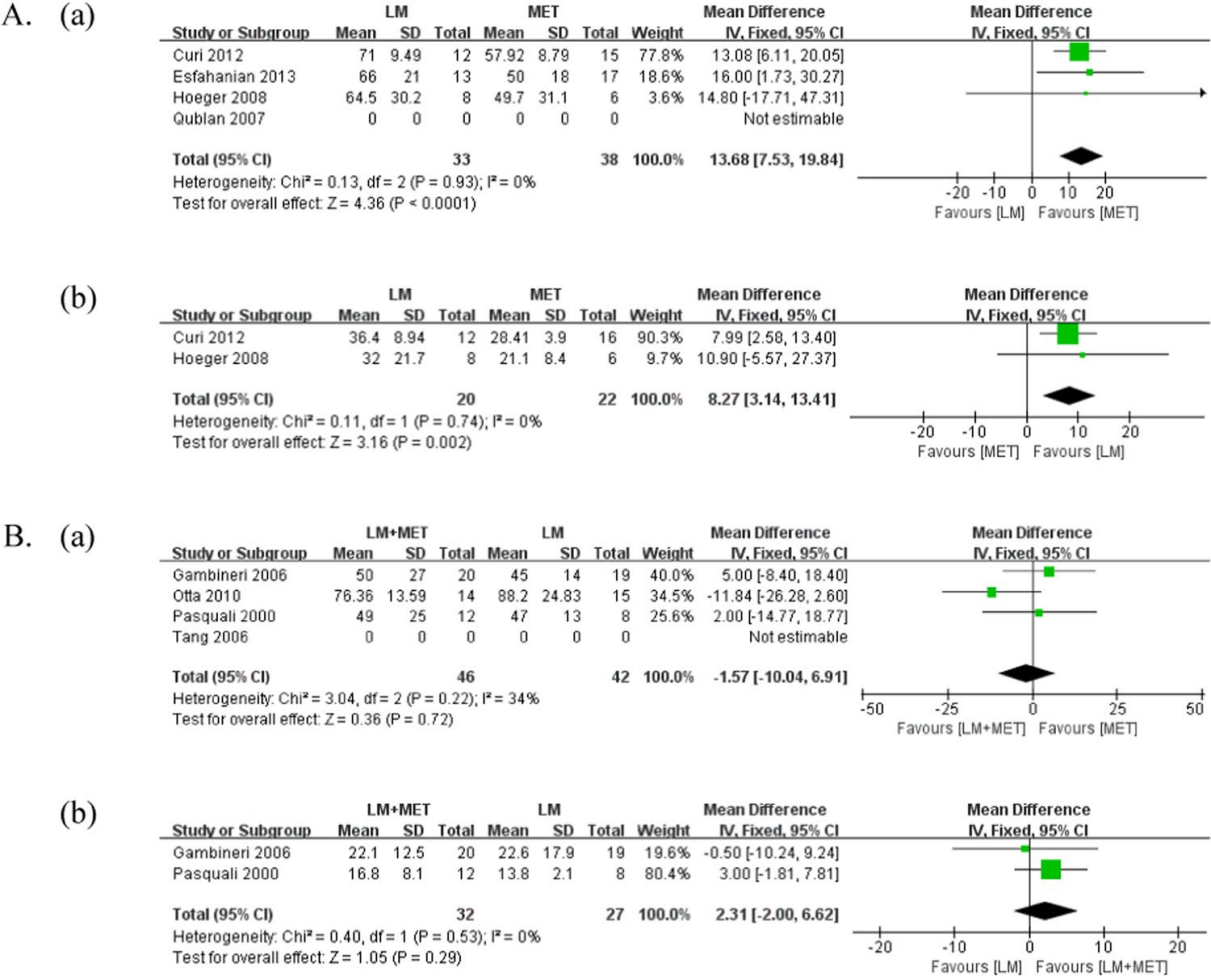
Meta-analysis of Androgenic parameters. (**A**) lifestyle modification versus metformin alone (**a**) Total testosterone (**b**) SHBG; (**B**) lifestyle modification + metformin versus lifestyle modification (**a**) Total testosterone (**b**) SHBG.

A comparison of androgenic parameters is presented in Fig. 6 and Supplementary 2. The total serum testosterone level was measured in five studies that compared LSM with metformin alone, and three studies were included for meta-analysis. Metformin alone significantly reduced the total serum testosterone level compared with LSM (p < 0.01; MD = 13.68). One out of five included studies reported a significant reduction in the total serum testosterone level with metformin alone, while two studies reported a significant reduction in both groups, and two studies reported no significant difference in either group.

Total serum testosterone levels were measured in seven studies that compared LSM + metformin with LSM, and three studies were included in the meta-analysis. There was no significant difference in the total serum testosterone levels between the groups (p = 0.72, MD = −1.57). Four out of six studies reported a significant reduction in the total serum testosterone level with LSM + metformin, while one study reported a significant reduction in both groups, and two studies reported no significant difference in either group.

#### Sex hormone-binding globulin

Serum sex hormone-binding globulin (SHBG) levels were measured in three studies that compared LSM with metformin alone, and two studies were included in the meta-analysis. LSM significantly increased serum SHBG levels compared with metformin alone (p < 0.01; MD = 8.27). One out of three included studies reported a significant increase in serum SHBG levels with LSM, whereas two studies reported no significant difference in either group.

Serum SHBG levels were measured in six studies that compared LSM + metformin with LSM, and two studies were included in the meta-analysis. There was no significant difference in serum SHBG levels between the groups (p = 0.29, MD = 2.31). Five out of six included studies reported significant differences in either group, and one study reported that LSM significantly increased serum SHBG level compared with LSM + metformin (p = 0.001).

#### Free androgen index

Free androgen index (FAI) was measured in two studies that compared LSM with metformin alone. However, we were unable to conduct the meta-analysis due to differences in the reporting data, such as the percent change (Hoeger *et al*.^23^). One out of two included studies reported a significant reduction in FAI with LSM, and one study reported no significant difference in either group.

FAI was measured in five studies that compared LSM + metformin with LSM. However, a meta-analysis could not be performed due to differences in the reporting data, such as the mean change (Ladson *et al*.^24^), percent change (Hoeger *et al*.^23^, Salama *et al*.^22^), and no standard deviation data (Tang *et al*.^21^). Three out of five studies reported a significant reduction in FAI with LSM + metformin, while one study reported a significant reduction with LSM, and one study reported no significant difference in either group.

## Discussion

To the best of our knowledge, the current study is the most updated and comprehensive systematic review and is the only study that analyzed subgroups based on the type of LSM. The principal finding of this study was that clinical outcomes, such as improvement in menstrual frequency and pregnancy outcomes, were not significantly different between LSM and metformin. Furthermore, the addition of metformin to LSM had no significant benefits in lowering BMI, while others found that LSM + metformin was associated with higher reduction in BMI in women with PCOS than LSM alone^18^. Additionally, we found that the majority of studies reported significant reductions in total serum testosterone levels with LSM + metformin compared with LSM alone, although there was no significant difference in the meta-analysis^18^ between the two groups.

Metformin alone does not improve the menstrual cycles in women who undergo LSM programs. In comparisons of LSM and metformin alone, our meta-analysis revealed that LSM tends to increase the number of patients who experience regular menstrual cycles more than metformin alone. Additionally, two of the four studies reported a trend of more patients experiencing menstrual regulation with LSM than with metformin alone. Although metformin was reported to improve the number of patients with regular menstrual cycles in a study by Tang^25^, LSM alone tended to increase the number of patients with improvement in menstrual patterns more than metformin alone in our systematic review.

We conducted two types of meta-analysis related to menstrual cycles between LSM + metformin and LSM. When comparing the number of patients with improvement in the menstrual patterns, there was no significant difference between LSM + metformin and LSM. LSM + metformin resulted in a significantly higher number of menstrual cycles over 6 months than LSM. This finding was consistent with the findings of a systematic review by Naderpoor^18^, which reported that LSM + metformin resulted in a higher number of menstrual cycles over 6 months than LSM alone. A systematic review by Tang^25^, which compared metformin with placebo, reported that metformin improved the menstrual patterns with an odds ratio (OR) of 1.72 (95% confidence interval [CI]: 1.14–2.61) in seven RCTs that included 427 participants. Although this meta-analysis indicated favorable results of metformin, it was limited due to the inclusion of only two studies in the meta-analysis. Additionally, the frequency of menstruation within 6 months, before and after the interventions, had significantly increased in both groups, but there was no significant difference in the improvement in the menstrual patterns.

Combining the above findings, we can state that there was no significant difference in the number of patients who experienced regular menstrual cycles between LSM + metformin and LSM. LSM alone appears to be sufficient in inducing regular menstrual cycles in women with PCOS; this suggests that the induction of spontaneous ovulation might be caused by the early corrections in the reproductive abnormalities. Additionally, it appears that metformin could have limited effects of reducing serum insulin concentration and inducing ovulation in non-obese women with PCOS, thus reflecting the heterogeneity in the pathogenesis of PCOS.

In terms of pregnancy rates, there was no significant difference between LSM and metformin alone. Additionally, the total pregnancy rates with LSM + metformin (8.7%) and LSM (2.7%) were not significantly different; this result is consistent with the results of the meta-analysis by Naderpoor^18^. Furthermore, the systematic review by Tang^25^ reported that metformin improved the clinical pregnancy rate compared with placebo (eight RCTs, 707 participants; OR: 2.31; 95% CI: 1.52–3.51). When comparing LSM with metformin alone, LSM tended to have a higher pregnancy rate than metformin (20% vs. 14.4%, 33.3% vs. 27.3%) although the difference was not statistically significant. Only a few studies have been performed with pregnancy rate as the primary outcome. Therefore, large, well-designed studies with pregnancy rate as the primary outcome are required to verify the current clinical outcomes.

LSM tends to have a high non-compliance of patients^23^, but due to the side effects of metformin, the dropout rates between LSM and metformin groups for the included studies were not significantly different. For the average dropout rate of 9 studies, LSM group was 19.5% (range: 5%~30%) and metformin group was 18.1% (range: 0%~44.4%), without any significant difference between the two groups (p = 0.836). 2 included studies statistically compared the dropout rate between LSM and LSM + metformin group, Tang^21^ reported that there was no significant difference in dropout rate between 2 groups (p = 0.23), and Ladson^24^ showed a similar result (p = 0.14).

In terms of side effects, there were 7 out of 13 studies reporting the side effects of metformin, and none of which reported the side effects of LSM. In metformin group, gastrointestinal side effects such as diarrhea, abdominal swelling, and flatulence were mainly reported^20,24,26^, and headaches, dizziness, and hair loss were also mentioned as side effects^24^. Ladson’s study comparing adverse events between the two groups reported that diarrhea and headaches were significantly more common in LSM + metformin group compared with LSM group^24^ (diarrhea, rate ratio = 3.2, p < 0.001; headache, rate ratio = 2.4, p = 0.003). Based on this result, the appropriate treatment should be selected carefully because of the side effects of metformin.

Both LSM and metformin have benefits in lowering BMI. LSM + metformin is known to be associated with lowering BMI in women with PCOS compared with LSM. Our results were different from those of a previous study, which demonstrated that adding metformin to LSM had no significant benefits in lowering BMI. The difference in the results is because we excluded five papers from the review process. We excluded three studies^19,24,27^ from Naderpoor’s study^18^ because their input data were baseline data instead of the post-intervention final data. Additionally, we excluded the study by Vanky^28^, which dealt with the effects of metformin in pregnant women with PCOS because our study only focused on women who wished to get pregnant. We also excluded the study by Hoeger^23^ because we could not find the results related to BMI; these caused the results to change conversely. The systematic review by Tang^25^ reported that there was no evidence of the effects of metformin on BMI (16 RCTs, 630 participants; MD = −0.05; 95% CI: −0.31–0.20), with an average treatment duration of 5.75 months and average dose of 1,500 mg of metformin, which is consistent with our results. Since all women included in this analysis underwent LSM, the benefits of metformin in lowering BMI may have been underestimated.

Metformin has additive effects on serum testosterone levels. Although we could not statistically confirm reduction in serum testosterone levels in the meta-analysis, four out of six studies reported significant reduction in total serum testosterone levels with LSM + metformin, while one study reported a significant reduction in both groups. These results suggest that metformin has additive effects on improvement in the parameters of laboratory hyperandrogenism. This result is consistent with that of several studies that have reported positive effects of metformin on hyperandrogenism. For instance, Tang^25^ demonstrated that metformin has additive effects on diet and exercise in improving the parameters of hyperandrogenism. Furthermore, in their systematic review, Tang^25^ reported that metformin reduced total serum testosterone levels with a MD of −0.60 nmol/L (14 RCTs, 610 participants; 95% CI, −0.73 to −0.48). Daily dosage and duration of metformin are not related to the magnitude of reduction in serum testosterone levels, and reduction in serum testosterone levels from metformin was classified into two categories^25^; in non-obese women, metformin significantly reduced serum testosterone levels, whereas it resulted in only marginally significant reduction in obese women. This is likely due to not well-controlled serum insulin concentrations in obese women with PCOS despite continuing metformin, which may eventually result in increased stimulation of androgen production from the ovaries, increase in the sensitivity of the pituitary gland to the effects of gonadotrophin-releasing hormone, and increase in steroid production from the adipose tissues^21,25,26,29,30^. The key finding of the study by Naderpoor^18^, which compared metformin alone with LSM, was that the metformin group demonstrated lower total serum testosterone levels after 6 months. When women with PCOS are treated with metformin, ovarian hyperandrogenism is attenuated and sustained, eventually resulting in lower serum testosterone levels^26^.

Metformin has an additive effect on fasting serum insulin levels but not fasting serum glucose levels. In comparisons between a combination of LSM + metformin and LSM, there was no significant difference in fasting serum glucose levels between the groups, while LSM + metformin significantly reduced fasting serum insulin levels compared with LSM. The results of the systematic review by Naderpoor^18^ were consistent with our results; both demonstrated no significant differences in fasting serum glucose levels. The systematic review by Tang^25^ found that the effects of metformin on fasting serum glucose levels were small (14 RCTs, 596 participants; MD = −0.15 mmol/L; 95 CI, −0.25 to −0.05), while metformin reduced the fasting serum insulin levels with a MD of −3.51 mIU/L (14 RCTs, 573 participants; 95% CI, −6.50 to 0.53). Metformin has been proven to reduce glucose absorption and hepatic glucose synthesis and increase insulin sensitivity by increasing peripheral glucose uptake with no significant direct effects on pancreatic insulin production. Insulin resistance in PCOS may arise as a result of defects in insulin signaling or receptor activity^31,32^, decreased insulin clearance due to the inhibitory effects of high serum testosterone levels^33^, and elevated adipose tissue, free fatty acids or cytokine production^34–36^. Furthermore, the mechanisms of insulin resistance in PCOS and metformin’s actions in improving the action of insulin are still largely unknown. The clinical efficacy of reduction in serum insulin levels in PCOS suggests that the reproductive abnormalities may be directly related to hyperinsulinemia rather than insulin resistance.

This systematic review represents the most updated and comprehensive analysis of the data on LSM and metformin to date. One limitation of this study is that some of these studies were small and included methodological weaknesses. The participants could not be blinded because of characteristics of the interventions. However, the outcomes including BMI, testosterone, FAI, menstrual cycles, fasting serum glucose, and fasting serum insulin are objective and were unaffected by the lack of blinding of participants. Second, the heterogeneous interventions made some of the comparisons difficult, making it challenging to draw conclusions. Third, the sample size of the included studies was not large enough. Fourth, a large-scale multicenter study of LSM + metformin is currently needed. Therefore, the follow-up duration in most studies was 6 months, and additional studies with longer follow-up periods are warranted. Lastly, there is a possibility of type I error due to the small number of studies included in the meta-analysis.

## Conclusion

Based on this systematic review, the clinical outcomes, such as improvement in the menstrual frequency and pregnancy outcomes, were not significantly different between LSM and metformin. LSM tends to have more benefits, except in terms of serum testosterone levels, than metformin alone. The effectiveness of LSM + metformin is limited to fasting serum insulin levels and menstrual cycles compared with LSM, and the addition of metformin to LSM resulted in no significant benefits in lowering BMI. Based on these results, we suggest selecting the appropriate treatment carefully while considering the side effects of metformin. If metformin is not indicated, LSM should be the primary recommendation in women with PCOS before prescribing metformin. A large-scale multicenter study of LSM + metformin is required to verify the currently controversial benefits and clarify the therapeutic role of this combination against PCOS.

## Methods

### Search strategy

We performed a systematic review to identify relevant articles that compared the effects of LSM with metformin alone on PCOS and the effects of LSM + metformin compared with those of LSM. We searched three English databases: Ovid-Medline (1946–December 2019), Ovid-EMBASE (1974–December, 2019), and the Cochrane Central Register of controlled Trials (Central). We designed strategies that included Medical Subject Headings (MeSH), such as “exp body mass index/, exp overweight/”, “exp infertility/, exp polycystic ovarian syndrome/, exp pregnancy/”, and “lifestyle modification, exp diet/, exp exercise/, exp weight loss/”.

### Eligibility criteria and study selection

To exclude irrelevant studies, two reviewers (KCH and LSH) independently screened the titles and abstracts, and a full-text review was subsequently performed. The selected studies were included based on the following inclusion criteria: (a) patients with PCOS; (b) comparative studies of LSM and metformin; and (c) studies that measured at least one outcome of interest. Review articles, abstracts, conference posters, and studies not in English or Korean were excluded.

### Data items and data collection process

The reviewers independently extracted the variables of interest from the selected studies using a data extraction form and reviewed the collected data twice to ensure accuracy. The primary endpoints were clinical outcomes, such as menstrual cycles and pregnancy rate, and the secondary endpoints were parameters such as weight loss and BMI; metabolic parameters, such as serum fasting glucose and insulin levels and HOMA-IR; and androgenic parameters, such as total serum testosterone and SHBG levels and FAI.

### Assessment of risk of bias

An assessment of risk of bias was also independently performed by two reviewers (KCH and LSH) using the Cochrane risk of bias (RoB) for RCTs. The Cochrane RoB for RCT assesses for selection bias, allocation bias, performance and detection bias, attrition bias, and reporting bias by classifying the studies as low, unclear, or high risk. All discrepancies were resolved by discussion with a third reviewer.

### Summary measures and synthesis of results

The statistical measures included ORs and MDs along with 95% CIs for dichotomous and continuous variables. The chi-squared test was used to assess the statistical heterogeneity between studies with significance set at p < 0.10, and heterogeneity was quantified using I^2^. Based on the degree of study heterogeneity, a fixed effect model was applied to calculate the summary measures, as appropriate. The publication bias was not assessable in these trials because this approach is generally appropriate when at least 10 studies are included in a meta-analysis. We conducted meta-analyses using Review Manager v5.3. Additionally, we examined the differences in the variables before and after the intervention for qualitative analysis to determine the reason of insignificant data in the meta-analysis using a two-tailed test of significance (p < 0.05).

## Supplementary information

Supplementary Table.
